# Confronting the complexities of antimicrobial management for *Staphylococcus aureus* causing bovine mastitis: an innovative paradigm

**DOI:** 10.1186/s13620-024-00264-1

**Published:** 2024-02-28

**Authors:** Shamsaldeen Ibrahim Saeed, Nor Fadhilah Kamaruzzaman, Noel Gahamanyi, Thi Thu Hoai Nguyen, Delower Hossain, Ivan Kahwa

**Affiliations:** 1 Nanotechnology in Veterinary Medicine Research Group, Faculty of Veterinary Medicine, Universiti Malaysia Kelantan (UMK), Pengkalan Chepa, Kelantan 16100 Malaysia; 2 Microbiology Department, Faculty of Veterinary Science, University of Nyala, PO Box 155, Nyala, Sudan; 3 Biology Department, School of Science, College of Science and Technology, University of Rwanda, P.O. Box 3900, Kigali, Rwanda; 4 Microbiology Unit, National Reference Laboratory, Rwanda Biomedical, P.O. Box 7162, Kigali, Rwanda; 5 Research Center for Infectious Diseases, International University, Vietnam National University, Ho Chi Minh City, Vietnam; 6 Department of Veterinary Medicine and Animal Sciences (DIVAS), Università degli Studi di Milano, Lodi, 26900 Italy; 7 Department of Medicine and Public Health, Faculty of Animal Science and Veterinary Medicine, Sher-e -Bangla Agricultural University (SAU), Dhaka, 1207 Bangladesh; 8 Udder Health Bangladesh (UHB), Chattogram, 4225 Bangladesh; 9 Department of Pharmacy, Faculty of Medicine, Mbarara University of Science and Technology, P.O Box 1410, Mbarara, Uganda

**Keywords:** Mastitis; therapy, *S. aureus*, Antimicrobial resistance, Alternative treatment

## Abstract

Globally, Mastitis is a disease commonly affecting dairy cattle which leads to the use of antimicrobials. The majority of mastitis etiological agents are bacterial pathogens and *Staphylococcus aureus* is the predominant causative agent. Antimicrobial treatment is administered mainly via intramammary and intramuscular routes. Due to increasing antimicrobial resistance (AMR) often associated with antimicrobial misuse, the treatment of mastitis is becoming challenging with less alternative treatment options. Besides, biofilms formation and ability of mastitis-causing bacteria to enter and adhere within the cells of the mammary epithelium complicate the treatment of bovine mastitis. In this review article, we address the challenges in treating mastitis through conventional antibiotic treatment because of the rising AMR, biofilms formation, and the intracellular survival of bacteria. This review article describes different alternative treatments including phytochemical compounds, antimicrobial peptides (AMPs), phage therapy, and Graphene Nanomaterial-Based Therapy that can potentially be further developed to complement existing antimicrobial therapy and overcome the growing threat of AMR in etiologies of mastitis.

## Introduction

Mastitis refers to an inflammatory condition of the udder in dairy animals. It is one of the more prominent dairy disease among lactating bovines, resulting in significant financial consequences for the dairy industry due to reduced yields of milk, raised early replacement and culling, and higher management and treatment expenses [1]. An estimated 19.7 to 32 billion US dollars are lost to mastitis each year in the dairy industry worldwide [2]. Mastitis has been associated with a variety of microorganisms, including bacteria, fungi, viruses, and algae [3]. It is reported that bovine mastitis can be caused by over 150 different types of bacterial species [4]. The major bacteria associated with mastitis are *Staphylococcus (S.) aureus*, *Streptococcus (S.) uberis, S. dysagalactiae, Escherichia (E.) coli, Klebsiella (K.) pneumoniae, and Pseudomonas (P.) aeruginosa, and Mycoplasma spp*. etc. [5]. Among them, *S. aureus* is the predominant pathogen associated with intramammary infection (IMI) due to its typical abundance in the udder skin teat microbiota. *S. aureus* sometimes enters through the teat tips and duct and colonize the inside of the udder [3]. Apart from being a mastitis etiology, *S. aureus* has been considered as a major human health hazard, particularly due to the recent development of methicillin-resistant *S. aureus* (MRSA). It has been reported that clonal complex (CC) 398 of livestock-associated MRSA (LA-MRSA) clonal complex (CC) 398 are also responsible for human infections [6]. The use of different antimicrobials is the widely used approach of treating any IMI in cattle. Nevertheless, there are certain drawbacks to this current approach of using antimicrobials due to the possibility of antimicrobial residues in milk, development of antimicrobial resistance (AMR), and low cure rate [7, 8]. Also, bacteria causing mastitis, particularly *S. aureus* cannot respond easily to therapy with antimicrobial agents due to the ability of the bacteria to enter and reside intracellularly within the mammary gland, providing additional challenges to the therapy [9]. The cellular invasion of *S. aureus* in udder establishes a reservoir that that promote subsequent re-infection [10], leading to a prolonged disease phase and recurring infections [11]. Moreover, recurrent and subclinical infections of IMI are also facilitated by the facultative survival of the *S. aureus* within cells [9]. Consequently, *S. aureus* remains protected from immune reaction within host and antimicrobial activity by the formation of biofilm and development of intracellular survivability [12]. This review aims at highlighting challenges of treatment of bovine IMI using conventional antimicrobial therapy and provides an overview of the alternative antimicrobials that can be used to complement existing therapy and, therefore, reduce the burden of AMR.

Antimicrobial Treatment for Mastitis Antimicrobials are widely prescribed in the dairy industry, primarily for the treatment of various infectious diseases. Among them, mastitis remains the most frequently treated ailment, estimated to account for twice the annual use of antibiotics in veterinary medicine [13, 14]. In addition to treating different diseases, antimicrobials are currently used for prophylaxis to prevent diseases in dairy animals [15].

The selection of antimicrobials for IMI in dairy cows is based upon the specific etiological agent responsible for the disease [13]. Various antimicrobials, including streptomycin, ampicillin, cloxacillin, penicillin, and tetracycline, have been applied for treating IMI, as outlined in Table 1 [16]. Along with other antibiotics, penicillin, aminoglycosides such as gentamicin and amikacin, and fluoroquinolones are widely used for IMI [13]. Cephalosporins including third generation (ceftiofur) and fourth generation (cefquinome) have also been used to bacterial infections including those causing mastitis [17]. The indiscriminate application of antimicrobials for the treatment and management of IMI significantly raises the probability of AMR in bacteria that have the potential to be transmitted to consumers through the food chain [18]. Apart from AMR, misusing antimicrobials negatively affects gut microbiota of dairy cows [17].

**Table 1:** FDA-approved antimicrobials for use in dairy cattle to treat mastitis (adapted from NMPF, 2020)

*Route of Administration*	*Antimicrobial class*	*Antimicrobial agents*	*Product name*	*Manufacturer*
Intramammary	Beta (β)-lactam Lincosamide	Penicillin G Amoxicillin Ceftiofur Cephapirin Cloxacillin Hetacillin Pirlimycin	Hanford’s/US Vet MASTICLEAR® Amoxi-Mast® SPECTRAMAST™ LC Today® Dariclox® Hetacin®K Pirsue® Sterile Solution	G.C. Hanford Mfg. Co Merck Animal Health Zoetis, Inc Boehringer Ingelheim Vetmedica, Inc Merck Animal Health Boehringer Ingelheim Vetmedica Zoetis, Inc
Injectable	β-lactam Tetracyclines Sulphonamide	Ampicillin Ceftiofur Ceftiofur Ceftiofur Penicillin GOxytetracycline Sulfadimethoxine	Polyflex® EXCEDE® EXCENEL® RTU EZ Naxcel® Sterile Powder Agricillin® Agrimycin 200 Di-Methox Injection 40%	Boehringer Ingelheim Vetmedica, Inc Zoetis, Inc Zoetis, Inc Zoetis, Inc Agri Laboratories, Ltd Agri Laboratories, Ltd Agri Laboratories, Ltd
Oral	Sulphonamide	Sulfadimethoxine	ALBON® Bolus	Zoetis, Inc
Topical	Tetracyclines Polymyxins	Oxytetracycline Polymyxin B	Terramycin® Ophthalmic Ointment with Polymyxin	Zoetis, Inc

## Challenges to antimicrobial treatment of *S. aureus* causing mastitis

### Antimicrobial resistance (AMR)

Antimicrobials are widely used in the dairy industry to treat and prevent mastitis. However, indiscriminate use of antimicrobials and not following the treatment regiments, have been found to be partially correlated to the raising the rate of AMR bacterial pathogens [19]. It is currently a global concern that widespread usage of antibiotics has resulted in the development of AMR bacteria to almost all antimicrobials and they are often referred to superbugs. The ability to transmit AMR bacteria along the food-chain is an additional challenge for the therapeutic management of infectious diseases in both humans and animals [20]. A variety studies reported AMR bacteria from bovine milk worldwide, especially those resistant to penicillin G [21]. Penicillin, a beta (β)-lactam antimicrobial, has been used extensively for curative and preventative treatment of dairy animals for over five decades which could explain an increased resistance to it [21]. Penicillin-resistant *S. aureus* was one of the first AMR bacteria reported in 1948 just a few years after the extensive manufacturing and use of penicillin [22]. There have been reports of AMR resistant bacteria in milk, with a significant proportion of *S. aureus* strains identified in both clinical mastitis cases and milk samples demonstrating resistance to β-lactam antibiotics, ranging from 60–90% [23–27]. This phenomenon of resistance can be attributed to the acquiring of the *mecA* gene, which is responsible for encoding the β-lactam-insensitive penicillin-binding protein (PBP2a or PBP2) [28]. The latter codes for a peptidoglycan transpeptidase enzyme that plays a role in the production of the cell wall when β-lactam antibiotics are present, allowing *S. aureus* to survive [29]. The rapid development of AMR in *S. aureus* is mediated by mutations, mobile genetic elements, or horizontal transfer of resistance genes [30]. Most horizontally acquired AMR is encoded by genes located on plasmids or transposons [28].The susceptibility of bacteria causing mastitis to antimicrobial treatment varies among different farms and regions depending on dairy production systems, management practices and legislation for the antimicrobial therapy, and the presence of AMR strains [21]. Globally, Africa, Asia, and Latin America are the leading regions where most of the resistance to antimicrobials (clindamycin, gentamycin, and oxacillin) have been reported [21]. In China, over 80% of *S. aureus* isolated from mastitis in cattle were resistance to penicillin and ampicillin while 50% of the isolates were resistant to erythromycin, aminoglycosides, and tetracyclines. In contrast, bacteria isolated from dairy in USA and European countries were reported to be resistant to less than 50% penicillin [31]. Surprisingly, the resistance rate of *S. aureus* isolates was much lower in Scandinavian countries including Sweden, Norway, and Denmark [31]. In Malaysia, our recent study demonstrated high resistance of *S. aureus* isolates to penicillin (46%), ampicillin (43.6%), oxacillin (31%), tetracycline (26%), and erythromycin (18%) [32]. The antibiotic resistance in mastitis causing isolates of other pathogens was also reported to be common though varied from antibiotic to antibiotic with highest rate found for sulfonamides, sulfamethoxazole, lincomycin and lowest for fluoroquinolones, and carbapenems [33, 34].

### Bacterial biofilm formation

Microbial biofilms are an additional challenge in treating infectious diseases [35]. Biofilms can be defined as microbial community adhered to the abiotic or biotic surface surrounded by a self–produced polymer matrix composed of proteins, polysaccharides, mineral crystals, and extracellular DNA [36]. Biofilms are developed through a sequential series of steps, commencing with the attachment of cell to surface, followed by adhesion between cells and surface and formation of extracellular matrix that protect the bacteria from being targeted by antimicrobial therapy, host defense systems and environmental stress [37]. The development of biofilms is considered to be a microbial protective mechanism that helps bacteria to escape from the host immune defense, antimicrobial actions and allowing them to survive in hostile environment [36]. Generally, planktonic cells are more affected by antimicrobials than biofilm embedded pathogens due to the impermeability of biofilm together with reduced growth rates and metabolic activities of biofilm residents [38–41]. Also, bacteria within a biofilm can express many chemicals and enzymes that may destroy antimicrobials [42, 43].

Biofilm‐related infections are particularly chronic and characterized by the persistence of microorganisms. The nature of biofilm infections may be linked to a specific group of cells residing within the biofilm structure referred to as "persister cells" [44]. In addition, the production of biofilms may possibly serve as a virulence factor related to IMI caused by *S. aureus*. Biofilm formation can enhance colonization and adherence of *S. aureus* in the udder, which involves attachment to the udder epithelium, proliferation and accumulation of cells in multilayers [45]. Thus, biofilm producing *S. aureus* can cause chronic infection in the udder, bringing an additional challenge for mastitis therapy. Moreover, the biofilm structure gives *S. aureus* additional protection against phagocytosis process of the immune system [36].

### Intracellular localization of bacteria

Many pathogenic bacteria can infiltrate and survive within the eukaryotic cells such as endothelial cells, fibroblasts, osteoblasts, and bovine mammary epithelial cells [46]. The intracellular environment offers a niche in which bacteria can continue to multiply or persist and hide from the host immune system [12]. Some bacteria are obligate intracellular including *Chlamydia spp*., and *Rickettsia spp.* while others (*Mycobacterium spp*., *Listeria monocytogenes*, *Salmonella spp.*, *Shigella spp.*, and *S. aureus*) are facultative intracellular bacteria [9, 47–53]. Facultative intracellular bacteria can live and grow either outside or inside the host cell, and they prefer to invade the host cell when they can benefit from the host cells [54, 55]. In contrast, the obligate intracellular bacteria cannot survive outside their host cell, so they strictly depend on host cells to live and grow. The host cell offers the essential source to support the growth of these bacteria [54]. Obligate intracellular bacteria cannot be grown in laboratories on culture medium. However, they can only grow in eukaryotic host cells such as animal hosts, embryonated eggs, and cell culture [53, 55].

**Fig. 1:**
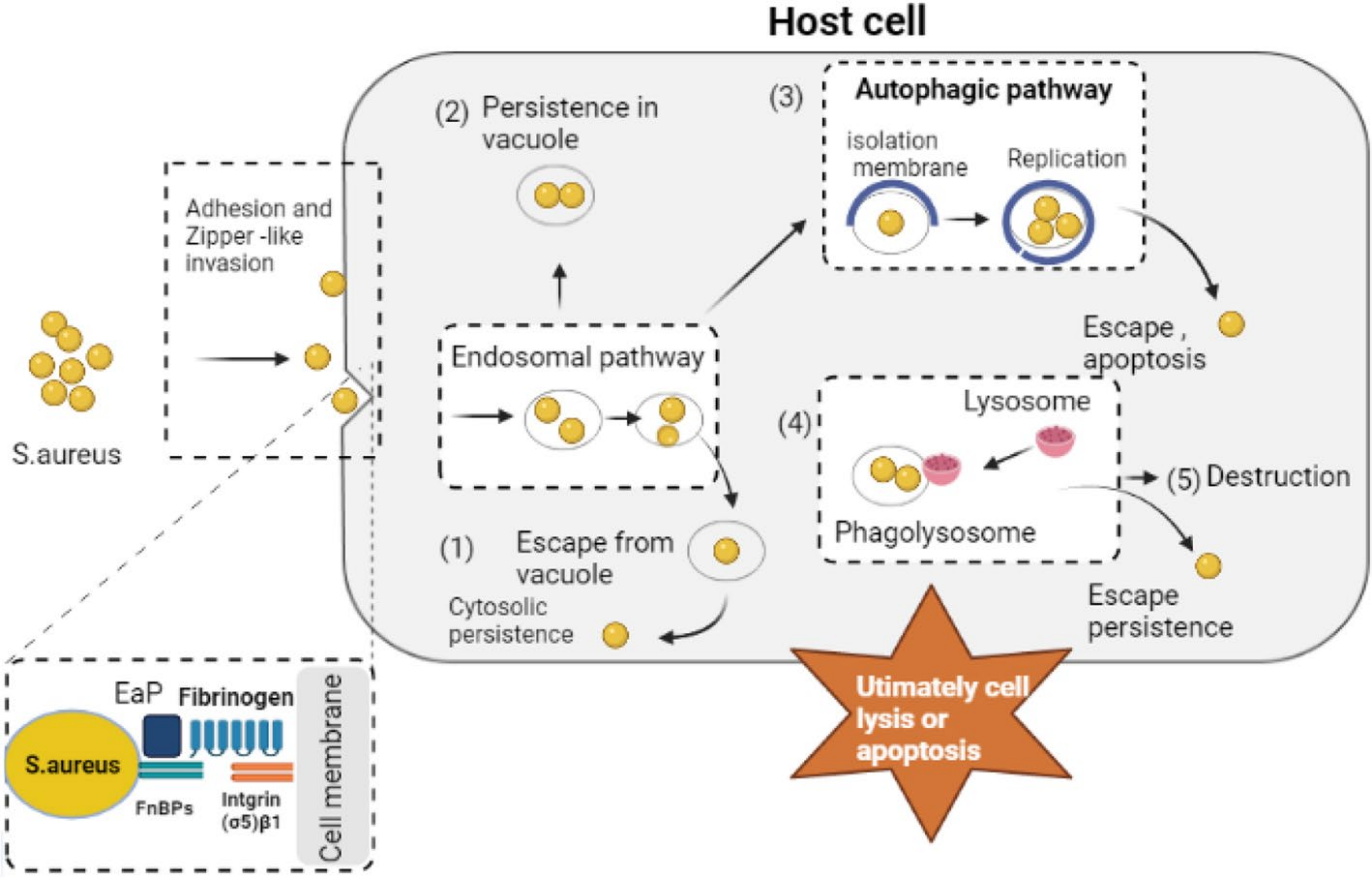
The process of infiltration of S. *aureus* to udder cells and its outcomes within the cells. The potential outcomes include (1) escape from the endosomal compartment, (2) persistence in vacuoles, (3) isolating in membrane, (4) escape from lysosome, and (6) destruction by lysosomal enzymes. Finally, cell lysis allows released *S. aureus* to infect new cells. The figure was drawn using Biorender.com

Intracellular bacteria can infiltrate the host cells using specific molecules represented by adhesion-function proteins, followed by invasion using the endocytosis pathway or zipper mechanism [56, 57]. In the case of *S. aureus* causing mastitis, the means of intracellular invasion occurs through a zipper uptake mechanism (Fig. 1). The process involves adhesion of bacteria to the surface of host cells, leading to the reorganization of the cytoskeleton. This rearrangement facilitates the movement of bacteria into host cells and survive and multiply within the acidic phagolysosome. Bacteria can also escape from the phagosome into the cytosol inducing cell death and bursts, subsequently entering the bloodstream to cause septicemia [58]. The intracellular localization results in a long term and persistent infection [59]. The treatment of bovine mastitis associated with intracellular *S. aureus* remains a challenge due to the poor ability of conventional antimicrobial agents to penetrate the host cells to reach the bacteria [60]. The primary barier for effective antibacterial therapy is distribution of antibacterials to specific regions inside the host. This process requires the crossing of host cell membranes either by diffusion or endocytosis [60]. Therefore, antimicrobial agents must possess the ability to pass the cellular barriers and subsequently enter the cytosol, where bacterial pathogens live. Some bacteria localized in highly acidic environments are also found in the lysosome and phagolysosome. This environment gives an additional protective barrier to the bacteria because many antimicrobials are ineffective in an acidic environment [61].

## Alternatives options for mastitis treatment

To overcome the challenge associated with the current antimicrobial therapy of bovine mastitis, it is essential to put effort into the discovery and advancement of alternative antimicrobial agents. Several antimicrobial replacements have been studied, suggesting the critical need for these antimicrobial-like compounds in sustaining animal health [5, 62]. Worldwide, many alternative antimicrobial approaches have been devised to tackle increasing rates of infections caused by AMR pathogens [63]. Several potential alternatives that show effectiveness in combating microbial infections include herbal antimicrobial substances, antimicrobial peptides, bacteriophages, and nanomaterials.

### Phytochemical antibacterial compounds in mastitis treatment

Several secondary plant metabolites have been reported to possess antibacterial properties against different pathogenic microorganisms; thus, they stand a good chance to be used as an alternative to the resisted antibiotics [64–66]. Phytochemical compounds,, exhibit antimicrobial activities by altering membrane permeability and disrupting the microbial membranes biosynthesis [67]. Besides having antimicrobial activity, phytochemicals are known to have effects on tumors, inflammation and can scavenge free radicals. have antitumor, anti-inflammatory, and antioxidant effects [68].Therefore, different phytochemical compounds offer a promising avenue for alternative therapy in combating *Staphylococcus aureus*-induced mastitis due to their multifaceted mechanisms of action and minimal side effects due to their ability to exert diverse pharmacological activities (Table 2). For instance, polyphenols like flavonoids and tannins exhibit potent antimicrobial effects by disrupting bacterial cell membranes and interfering with essential enzymatic processes. Moreover, certain phytochemicals, such as alkaloids and terpenoids, can inhibit bacterial biofilm formation, which is crucial for *S. aureus* persistence and virulence [65, 69]. Additionally, the anti-inflammatory activity of phytochemicals helps alleviate the symptoms associated with mastitis, such as swelling and pain, while also supporting the immune system in combating the infection [70]. Importantly, phytochemicals offer a natural and sustainable approach to mastitis treatment, minimizing the risk of antibiotic resistance development and environmental contamination associated with conventional therapies] [71, 72].Thus, integrating phytochemical compounds into mastitis management protocols holds great promise for improving treatment outcomes and reducing reliance on antibiotics, addressing public health and animal welfare considerations.

**Table 2:** Summary of antimicrobial activity of phytochemical compound against bacteria associated with mastitis

Classes	Sources	Phytochemical	Bacteria	References
Phenolic compound	*Eucalyptus globulus* Labill, and *Juglans regia*	Eucalyptol, globulol, and aromadendrene	*S. aureus*	[69, 73]
Phenolic compound	*Ocimum tenuiflorum*	Linalool, eugenol, methylchavicol, methylcinnamat, linolen, ocimene, and pinene,	*S. aureus, S. agalactiae, and E. coli*	[70]
Phosphoric acid	*Melaleuca alternifolia*	Terpinen-4-ol Sabinene, α-Terpinene, Limonene, p-Cymene, α-Terpineol, Aromadendrene, and Globulol	*Staphylococcus spp., Streptococcus spp., E. coli, and K. pneumoniae*	[71]
Phenylpropanoid	Cinnamon oil	Cinnamaldehyde, eugenol, cinnamic acid, and cinnamate	*S. agalactiae*	[72]
Terpenoids	*Ocimum basilicum* and *Cymbopogon citratus* (lemongrass)	Linalyl acetate, and Geranial,	*S. aureus, and E. coli*	[74]
Terpenoids	Olive leaf extracts, olive, and its oil	Betulinic acid, rotundic acid, amyrin, saponins, Oleanolic acid, ursolic acid, ginsenoside, gypenosides, and tirucallane-type of Eurycoma longifolia	*S. aureus, and P. aeruginosa*	[75–77]
Terpenoids	*Melaleuca alternifolia*	Terpinene-4-ol	*S. aureus*	[78]

**Fig. 2:**
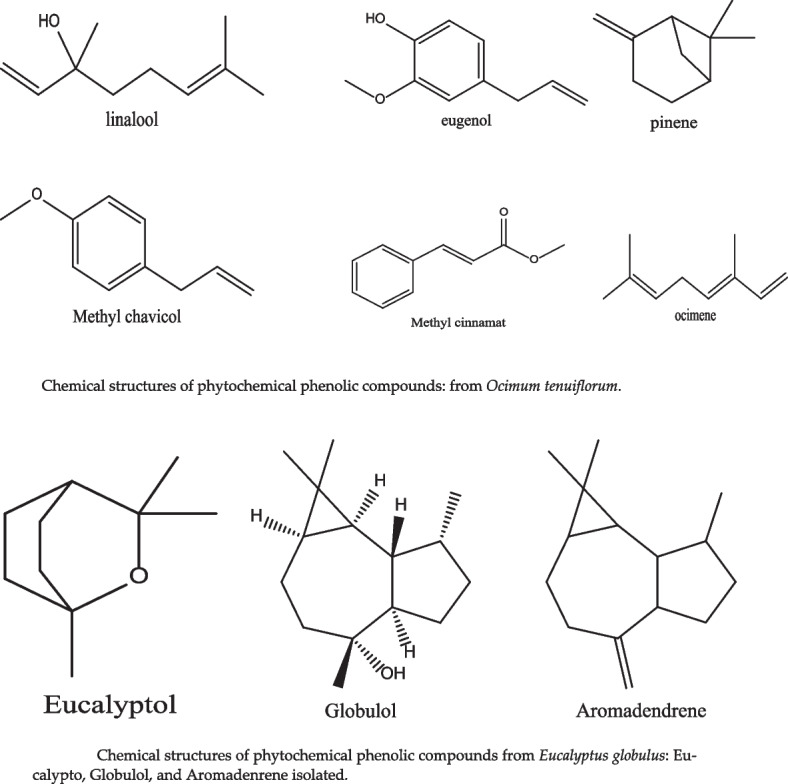
Chemical structures of phytochemical phenolic compounds from *Eucalyptus globulus*: Eucalypto, Globulol, and Aromadenrene isolated

**Fig. 3:**
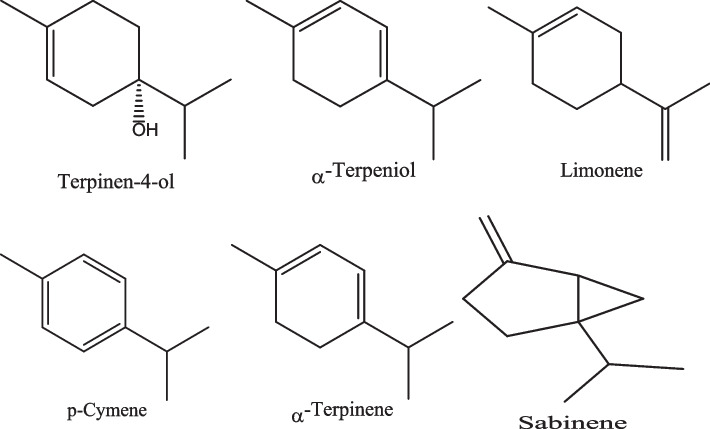
Chemical structures of phytochemical phenolic compound: Terpinen-4-ol, α- Terpeniol, Limonene, p- Cymene, α- Terpinene and Sabinene isolated from *Melaleuca alternifolia*

Several studies have reported the efficacy of phytochemical compounds for mastitis treatment targeting the broad-spectrum bacteria commonly resistant to mastitis for instance. Srichok et al. [70], carried out the antimicrobial and anti-inflammatory properties of extracts derivedfrom *Ocimum (O.) tenuiflorum* (Fig. 2). Additionally, the study investigated that potential interactions between *O. tenuiflorum* extracts and antimicrobial medications in relation to their efficacy against major IMI-causing pathogens including *S. aureus*, *S. agalactiae*, and *E. coli*. The *O. tenuiflorum* extract showed antimicrobial activity *S. aureus* and *S. agalactiae* (minimum inhibitory concentrations (MICs): 3.9–31.2 µg/mL and minimum bactericidal concentrations: (MBCs): 15.6-500 µg/mL) in this study. Moreover, there were identified synergistic effects when *O. tenuiflorum* extract was combined with β-lactam antibiotics, particularly penicillin or amoxicillin-clavulanic acid. Additionally, the extract showed a substantial reduction in the production of many inflammatory markers, including IL-6, TNF-α, IL-1β, iNOS, COX-2, and PGE2. This study suggested the effectiveness of the extract against the bacteria which is known to cause mastitis, hence potentially lowing the antimicrobial doses and minimizing anti-inflammatory responses [70]. Hase et al. [79], assessed the efficacy of topical herbal sprays and Mastilep gel (non-antibiotic polyherbal gel) against bovine subclinical mastitis. The active ingredient for both treatments is obtained from different plants including *Cedrus deodara, Curcuma longa, Glycyrrhiza glabra and Eucalyptus (E.) globulus*, known for their antimicrobial, and antiinflammatory properties. *E. globulus* contains different chemical compounds such as Eucalypto, Globulol, and Aromadenrene (Fig. 3). The study revealed that the application of the herbal spray and Mastilep gel significantly reduced the somatic cells and eliminated the bacteria causing mastitis within five days of application. Consequently, cure the mastitis compared to untreated group [79].

In another study, Cordeiro et al. [78] investigated the antimicrobial and antibiofilm properties of terpinen-4-ol derived from *Melaleuca (M.) alternifolia* against *S. aureus* isolated from mastitis (Fig. 3). The study findings indicate that terpinen-4-ol exhibits potent bactericidal and antibiofilm properties against all strains of *S. aureus*, with 0.25% (v/v) MIC, and 0.5% (v/v) MBC. This phytoconstituent is hypothesized to exert its mode of action by interruption of bacterial cell wall formation, with PBP2a being identified as one of its specific targets. This study suggests the potential use of the essential oil of *M. alternifolia* for treating bovine mastitis.

### Antimicrobial peptides (AMPs)

**Fig. 4:**
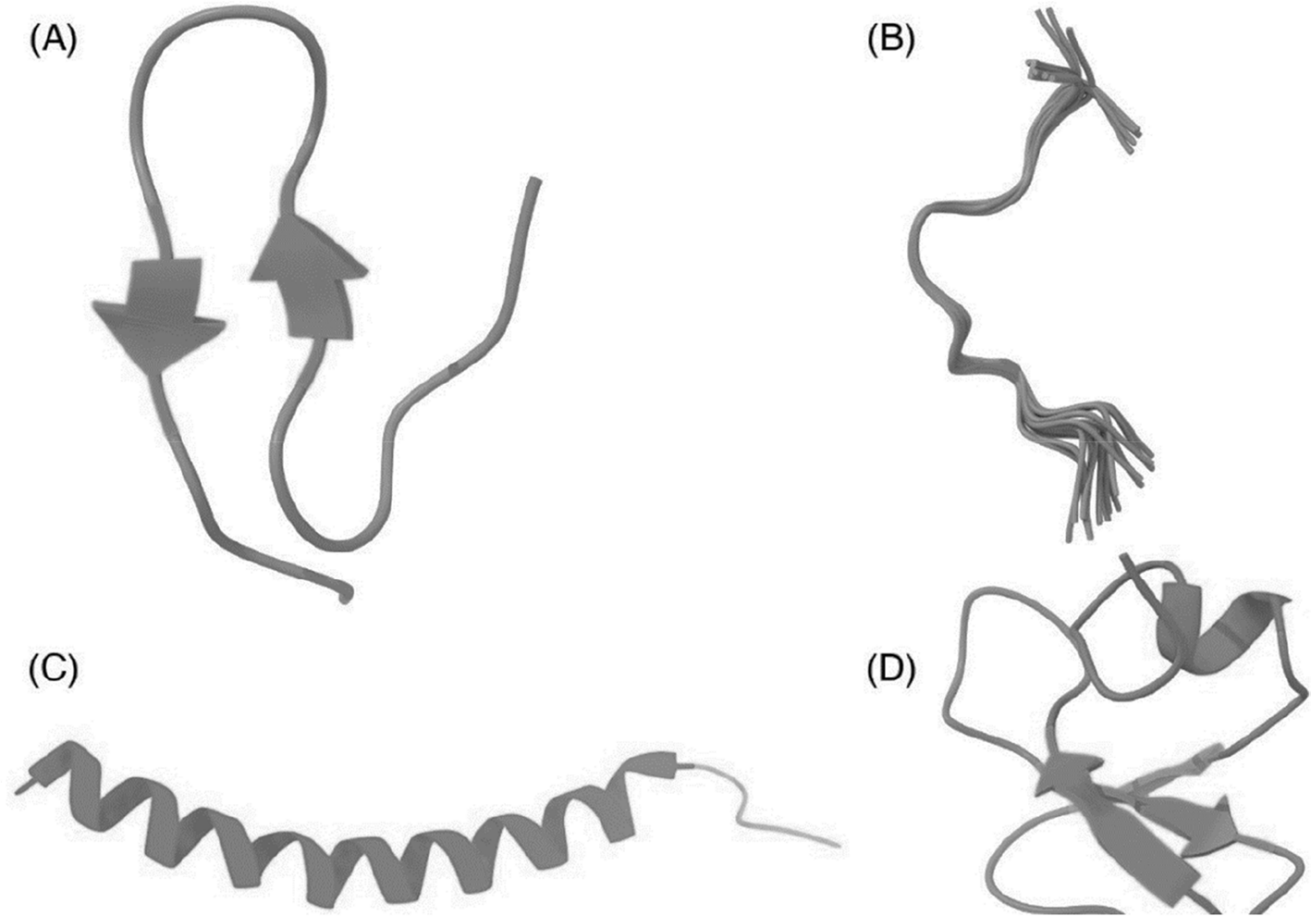
Structure of antimicrobial peptide (AMPs): **A** β-sheet, **B** Linear, **C** α-helical, and **D** combined structure. The figure was created using UCSF Chimera (http://www.cgl.ucsf.edu/chimera) The mechanisms underlining the AMPs antimicrobial proprieties is believed to be due to cell membrane disruptions. There is four mechanisms for AMPs membrane distribution has been identified including “Barrel stave”, “toroidal pore”, “carpet”, and ‘’aggregate’’ as describing in our previous study [62] (Fig. 5). Besides the damaging membranes, AMPs can kill bacteria by targeting and inhibiting the biosynthesis of proteins, nucleic acids, and essential enzymes required and involved in vital biological pathways and ultimately lead to cell lysis. The mechanisms for intracellular AMPs are summarized in Fig. 6. The antimicrobial activity of AMPs is particularly linked with its corresponding amino acid composition and physicochemical characteristics [81]. In addition to their direct antimicrobial activity, AMPs possess immunomodulatory properties, which stimulate the immune reaction of the host animal. By stimulating the functioning of immune cells and enhancing their functionality, AMPs contribute to a higher and effective protection against bacterial invaders [84, 86]. In an era of increasing antibiotic resistance, AMPs are emerging as a potent and promising therapeutic alternative. The AMP study demonstrates a remarkable advancement in the development of simple and accountable solutions in the prevention of S. aureus associated mastitis in dairy cattle [83–85]

AMPs are positively charged, amphiphilic, oligopeptides consisting of 10–50 amino acids [80]. This characteristic enables AMPs to adhere to and infiltrate the bacterial cell wall bilayer, resulting in the formation of pores through mechanisms known as "toroidal-pore," "barrel-stave," and "carpet”. Consequently, this process leads to the leakage of intracellular contents [81]. They come in a variety of structural forms including helical to linear and β-sheet structures (Fig. 4) [82].

AMPs have been tested against a variety of major mastitis causing pathogenic bacteria that are shown in Table 3. Both naturally occurring and artificially synthesized AMPs demonstrated potent and broad-spectrum antimicrobial actions against a wide range of major bacteria responsible for IMI. Tomasinsig et al. [83] reported in their study that cathelicidins, a class of peptides derived from bovine sources, including BMAP-27, BMAP-28, Bac5, and indolicidin, had a wide range of effectiveness (MIC = 0.5–32 µM) against a majority of bacterial isolates [83]. Shah et al. [84] examined the antimicrobial and antibiofilm activity of Polybia MP-1 (Mastoparan) peptide derived from the venom of the vespid wasp *Polybia paulista* against multi-drug resistant *P. aeruginosa* from bovine mastitis. The Polybia MP 1 demonstrated efficacy against tested pathogens with MICs of 75 µM and MBCs of 150 µM, according to the study's findings. Furthermore, Polybia MP-1 demonstrated very low to moderate hemolytic activity against red blood cells (RBCs) of goat, cow, and buffalodue to its strong membrane selectivity [84].

Cao et al. [85] tested the efficacy of AMP Nisin for the treatment of clinical form of bovine mastitis in Hangzhou, Zhejiang Province, China. The study found that, both nisin and gentamicin have great efficacy against mastitis, with cure rate estimated to be 90.2% and 91.1%, respectively. The bacterial culture and somatic cells analysis revealed no significant difference between the two groups. This observation indicated that nisin peptide is as effective as gentamicin in treating mastitis. Furthermore, 35.3% *S. aureus* isolates showed resistance to while no resistance was recorded fornisin [85]. Nisin is currently approved for clinical usage while some of its derivatives are at the advanced stages in clinical trials.

**Fig. 5:**
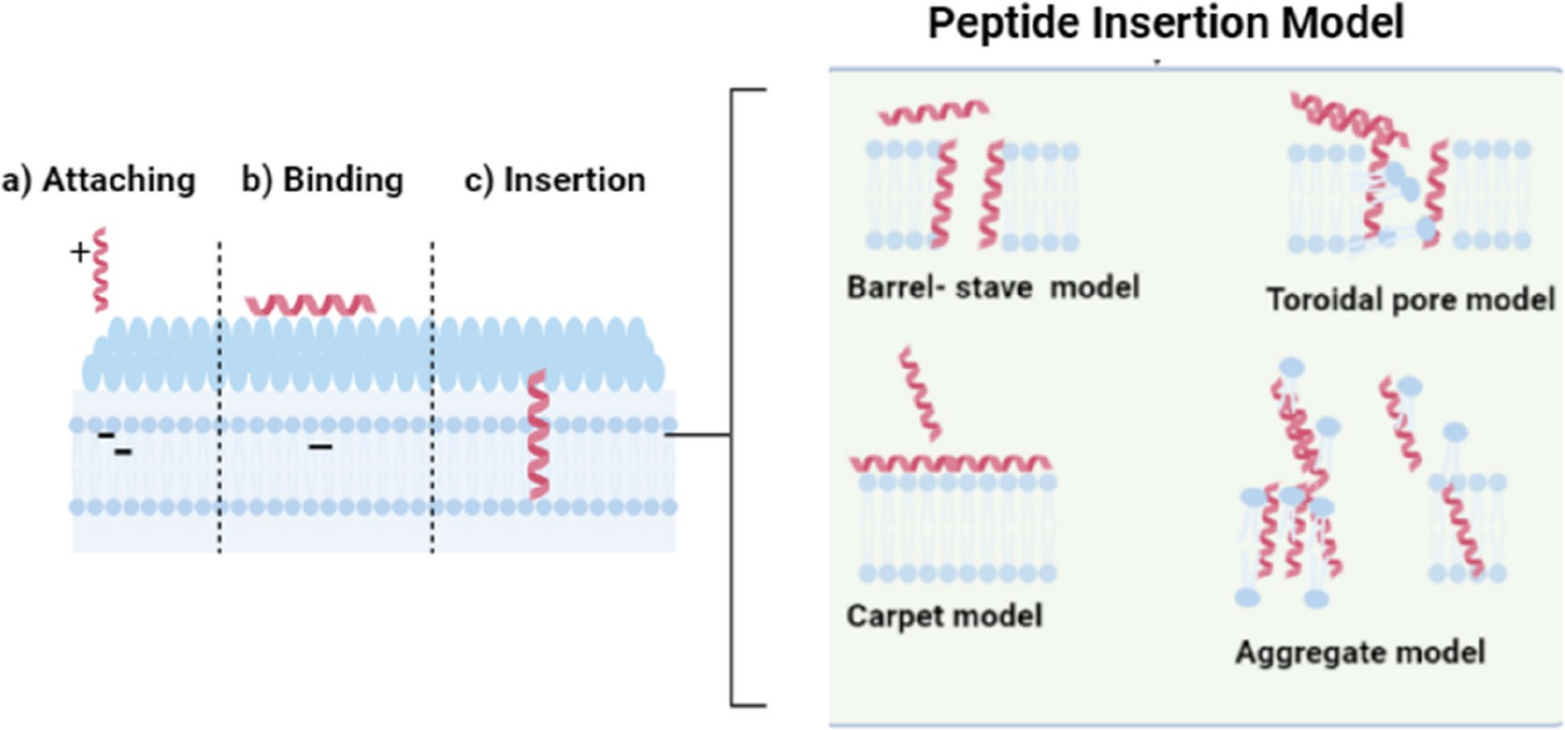
Mechanisms of action between peptide and bacterial cellular membrane. The image was created using BioRender.com and based in our previous work [62]

**Fig. 6:**
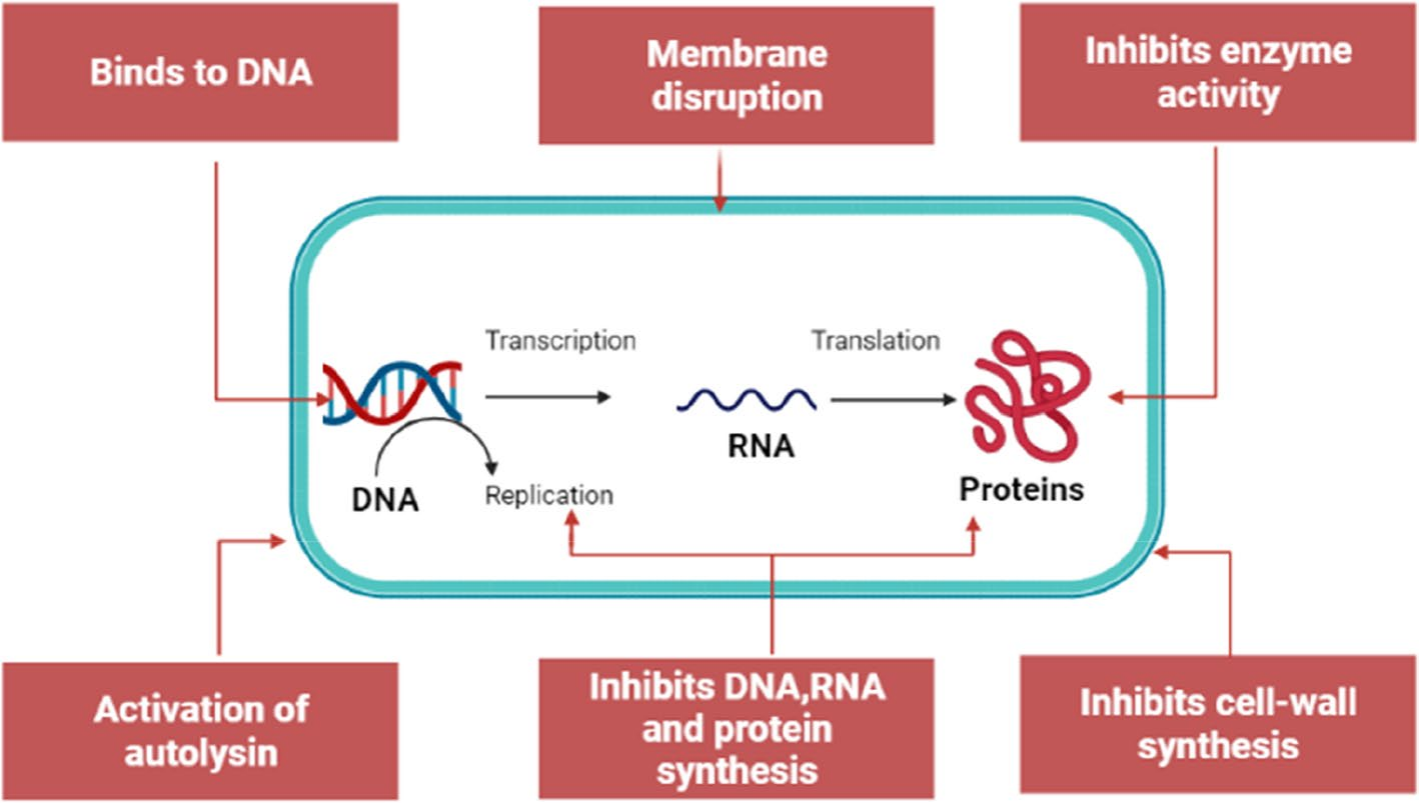
Mechanism for intracellular antimicrobial peptides activity. The image was created using BioRender.com and based in our previous work [62]

**Table 3:** Antibacterial efficacy of the peptide-based antimicrobial compound against major bovine mastitis causing pathogenic bacteria

Antimicrobial peptides	Bacterial species	Minimum inhibitory concentrations(µM)	References
Plectasin	*S. aureus*	3–6	[51]
Polybia MP-1 (Mastoparan)	*P. aeruginosa*	75	[84]
Nisin	*S. aureus*	> 32	[87]
Indolicidin	*E. coli*	4	[83]
	*K. pneumoniae*	4–8	
	*S. aureus*	2–8	
	*S. epidermidis*	1–2	
	*S. uberis*	1–2	
	*S. agalactiae*	1–2	
Fungal defensin-like peptide-P2	*S. dysgalactiae*	0.23–0.46	[88]
Cathelicidins Bac5	*E. coli*	0.5–1	[83]
	*K. pneumoniae*	1–4	
	*S. aureus*	> 32	
	*S. epidermidis*	1–2	
	*S. uberis*	16–32	
	*S. agalactiae*	4–6	
Cathelicidins BMAP-27	*E. coli*	0.5–4	[83]
	*K. pneumoniae*	1	
	*S. aureus*	4–8	
	*S. epidermidis*	0.5–1	
	*S. uberis*	4	
	*S. agalactiae*	4	
Cathelicidins BMAP-28	*E. coli*	2–8	[83]
	*K. pneumoniae*	1–2	
	*S. aureus*	2–4	
	*S. epidermidis*	1–2	
	*S. uberis*	2–32	
	*S. agalactiae*	2	

### Bacteriophage therapy

**Fig. 7:**
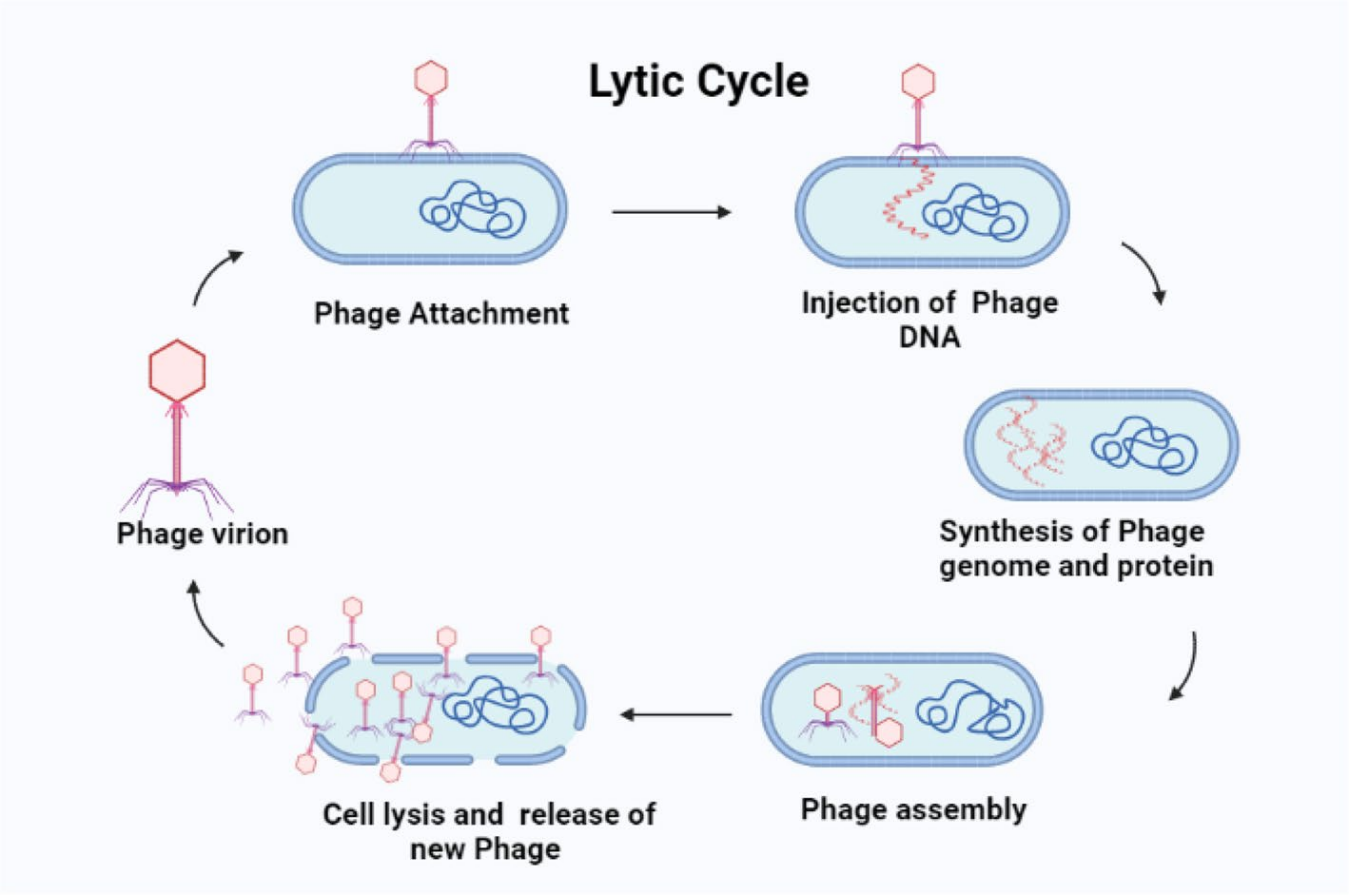
Mechanisms of action for bacteriophage antimicrobial therapy. Image represents the schematic diagram of developmental cycle of lytic bacteriophage. The figure was created by using BioRender.com

Viruses known as "phages," or bacteriophages, invade and multiply within bacteria and occasionally cause bacterial death [89]. Bacteriophages therapy has been suggested as a highly promising alternative to antibiotics because of its characteristics, which include high specificity, low toxicity, antibacterial activity, affordability, and the capacity to proliferate at the infection site [89]. The two main biological cycles of bacteria that phagophages can disrupt are the lytic cycle (phage DNA survives as an independent entity within the bacterial cell, undergoing replication independently from the host bacterial DNA, and subsequently causing lysis of the host cell to liberate newly formed phage components.) and the lysogenic cycle (phage DNA integrates into the host genome) [89] (Fig. 7).

Phages are specific in binding receptors of bacterial cells implying that they cannot infect human or animal cells including microbiota [90]. The main concern with phage therapy is associated with immune response to bacteriophages which can decrease their activity against bacterial pathogens [91]. Several studies have reported promising safety and efficacy of Phage therapeutic toward various pathogens associated with mastitis. More information on phage efficacy toward *S. aurues* associated with mastitis is presented in Table 4. For instance, Teng et al. [92] mentioned that phage 4086–1 had an outstanding efficacy against *S. aureus*-induced mastitis in a mouse model and could be a promising drug in treating mastitis. Another study using a murine model for bovine mastitis confirmed that the quantity of phage cocktail remained high in intramammary gland and did not spread [93]. However, the efficacy of phage in treating S. aureus-induced mastitis was reported to be limited under the treatment conditions studied (36 h vs 5 days) [93]. Also, phage therapy increased somatic cell count (SCC) in healthy quarters and the degree of inflammation may affect the amount of free phage available [93]. A recent systematic review reported that 13 clinical trials with phage therapy were safe [90].

Using murine mastitis and Galleria mellonella models, Ngassam-Tchamba et al.'s recent study [94] assessed the effectiveness of lytic phage on *S. aureus* producing bovine mastitis in vitro and in vivo. In the study, ten *S. aureus* isolates—five of which were methicillin-resistant and the other five of which were methicillin-sensitive—isolated from bovine mastitis were subjected to tests using four lytic bacteriophages: Rufus, Remus, ISP, and DSM105264. According to the data obtained, *S. aureus* isolates can be lytically attacked *in-vitro* by Romulus, Remus, and ISP. At the fourth day post-inoculation (DPI), a larval survival rate of less than 50% was noted in the groups treated with three phages *in-vivo* and infected with methicillin-sensitive *S. aureus* isolates. This finding implies that phage may be a useful treatment for mastitis [94]. Huijun Geng et al. [95] found a combined therapy of two lytic bacteriophages, vBSM-A1 and vBSP-A2. He demonstrated that this combination has a great therapeutic potential for mastitis treatment after significantly improving mastitis pathology and decreasing bacterial counts in mice with induced mastitis [95].

Guo et al. [91] found that three lytic phages SYGD1, SYGE1, and SYGMH1 collected from sewage of dairy farm were able to cure mastitis caused by multi-drug resistant *E. coli*. The administration of three phages cocktail significantly reduced the somatic cells, CFU/ml of bacteria, and inflammatory factors, leading to recovery from bovine mastitis, and achieved the same effect as antimicrobial therapy [91].

**Table 4:** Summary of some potential phages cocktails for treating S. aureus associated mastitis cases

The Phage cocktail	Phage sources	Bacteria spp	Bacteria resistance	Therapeutic efficacy	References
SAML-4 SAML-12 SAML-150 SAML-4229 SATA-8505	Commercial (StaphLyse™)	*Staphylococcus aureus (S. aureus)*	MRSA, MSSA, and VISA	The Phage reduced 92.7% and 100% of *S.aureus* at a titter of 2 × 10^4^ PFU/mL and 1 × 10^9^ PFU/mL respectively The phase was stable at 37 °C for 24 h and one week at 4 °C	[93]
Romulus Remus ISP	Sewage water	*S. aureus*	MRSA, MSSA	The phage has showed bactericidal activity toward *S. aureus* in vitro The three-phage reduced 50% of larvae survival rate at 4 days after infected with a methicillin-sensitive S. aureus in vivo Partial recovery of the mouse mastitis was recorded in days after infected and treated with ISP phage in vivo	[94]
Phage ATCC 23361 BP39	Commercial (Phage Lux)	*S. aureus*	MDR	The phage cocktail was examined in raw milk and in TSB broth with the addition of IgG as a potential suppression of phage activity after 4 h of bacterial multiplication The phage had significantly reduced CFU of *S. aureus* in both in raw milk and in TSB, with no significant impact with adding IgG to the culture	[96]
The Phage cocktail	**Phage sources**	**Bacteria spp**	**Bacteria resistance**	**Therapeutic efficacy**	**References**
*S. aureus* phage 4086–1,4086–2, 4086–3, 4086–4, and 4086–6,	Milk samples from mastitis cows	*S. aureus 4086* *S. aureus ATCC 43,300 Staphylococcus xylosus 17* *Micrococcus luteus 26,003* *Staphylococcus saparophytics 17 Staphylococcus saparophytics E4 Staphylococcus saparophytics X4 Staphylococcus haemolyticus 13*	MDR	The survival rate of *S. aureus* was inhibited after treating by phage 4086–1 The phage has anti-inflammatory effect by decreasing the concentration of TNF-α and IL-6	[92]
vB_EcoM_SYGD1 (SYGD1), vB_EcoP_SYGE1 (SYGE1), vB_EcoM_SYGMH1 (SYGMH1),	Sewage samples collected from dairy farms	*E. coli*	MDR	The three phages showed bactericidal activity against *E. coli* Reduced the somatic cells and inflammatory cells was recorded after treated with phages The phages were stable under different temperature and pH	[91]
vBSM-A1 and vBSP-A2	Sewage samples collected from dairy farms	*S. aureus*		Both phages have lytic activities against tested bacteria A significant recovery was reported in mice induced mastitis, and reduced bacteria count after treated with these phage’s in vivo	[95]

*Abbreviations* used in this table are: *MSSA* Methicillin-sensitive Staphylococcus aureus, *MDR* Multidrug resistance, MRSA Methicillin-resistant Staphylococcus aureus, *ISP* Intravenous staphylococcal phage, *VISA* Vancomycin intermediate Staphylococcus aureus

### Graphene nanomaterial-based therapy

**Fig. 8:**
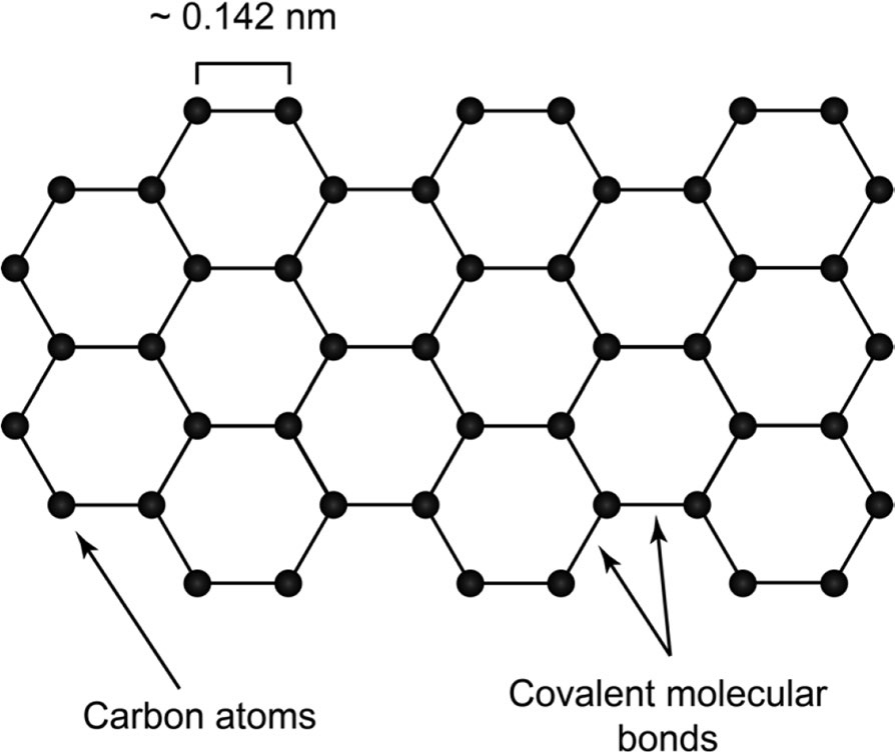
Chemical structure of graphene nanomaterials

Graphene is a two-dimensional carbon-based nanomaterial (CBNMs) that originated from graphite (Fig. 8). It was successfully isolated from graphene in 2004 by Novoselov et al. [97]. Graphene oxide (GO), reduced graphene (rGO), and graphene composite with other nanomaterial have been tested for its antimicrobial properties toward various pathogens including bacteria, yeast and parasite [98, 99]. Graphene antimicrobial activities are highly attributed to the physical characteristics, (size, sheet layers, shape, the surface modification, agglomeration, and dispersion) [100]. These physical characteristics influence the level of interaction of graphene with pathogens to demonstrate the antimicrobial activities.

**Fig. 9:**
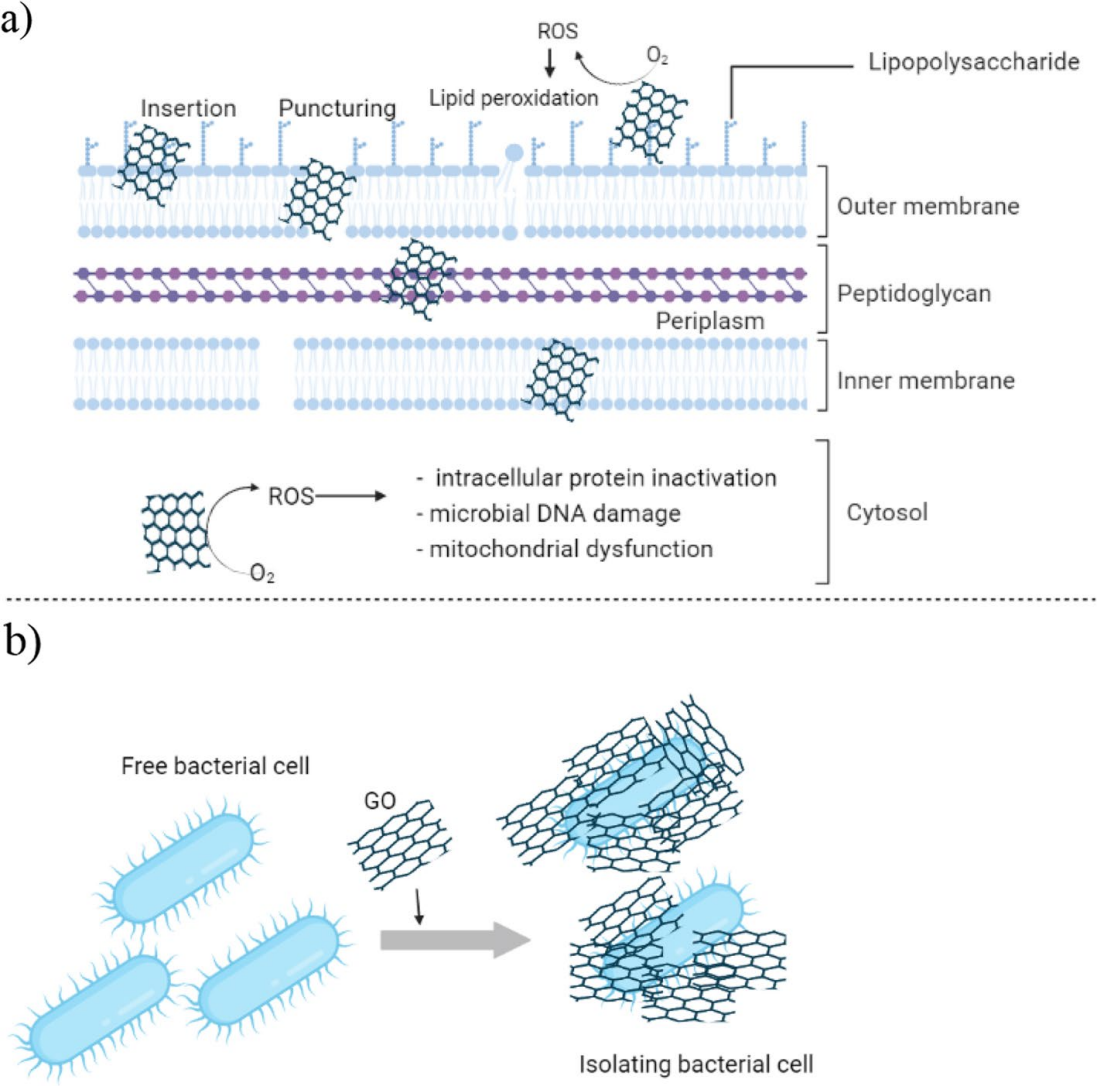
Antibacterial mechanisms of graphene oxide (GO). **a** Sharp edge effect and oxidative stress that can lead to membrane damage. ROS generation by GO causes DNA damage, protein inactivation and mitochondrial dysfunction of the bacteria. **b** trapping of the bacterial cells and isolating from nutrient. The figures were created by using BioRender.com

Graphene, GO, and rGO are believed to exhibit their antimicrobial activities due to several mechanisms such as i) the presence of sharp edges on GO surfaces could induce physical damage to the bacterial cell wall, thus causing the leakage of cellular components and the death of microbe [100]; ii) the large surface area of GO sheet can trap bacteria, isolating them from the environment and delaying bacterial growth and nutrient access [101]; iii) GO can induce oxidative stress (OS) leading to intracellular protein inactivation, microbial DNA damage, and mitochondrial dysfunction followed by the necrotic or apoptotic process and resulting in bacterial inhibition and death [100]. Figure 9 illustrate the mechanism of antimicrobial activities of Graphene- based nanomaterials antimicrobial activities.

Several in vitro studies on graphene antimicrobial properties have shown great bactericidal activity against pathogenic bacteria causing mastitis. Thus, suggesting that graphene and its derivatives have the potential to be further tested and developed as an alternative antimicrobial treatment for mastitis. Table 5 summarises graphene antimicrobial activities against a range of pathogens isolated from bovine mastitis.

The recent study by Vimalanathan et al. [102] demonstrated the antimicrobial activity and cytotoxicity of GO and thiourea-reduced oxide (T-rGO) nanosheets against E. coli isolated from mastitis and human prostate cancer cells. Both T-rGO and GO showed good antibacterial activity against E. coli mastitis. The growth of E. coli was reduced up to 89.8% and 87.7% after treatment with both GO and T-rGO, respectively. The antibacterial efficacy of T-rGO was slightly higher than that of GO. Furthermore, the production of hydroxyl radicals and ROS was increased following the treatment, and the DNA was harmed because of OS, causing laddering [102].

Our recent study has investigated the antimicrobial and antibiofilm activity of GO against S. aureus isolated from bovine mastitis, GO was found to be effective against extracellular and intracellular forms of S. aureus. GO at a 200 µg/mL reduced 90% of bacterial cells viability for all tested isolates. Also, GO at 100 μg/mL reduced between 30–70% of S. aureus biofilm mass, suggesting GO ability to disrupt the biofilm structure. the toxicity was recorded at a concentration higher than 1000 μg/mL, which is higher than the concentration needed to inhibited the bacteria growth [103]. Despite the antimicrobial properties of Graphene – based materials several other studies measured GO toxicity towards the following cell line, human breast cancer, ovarian cancer, HeLa and mouse embryonic fibroblast. Briefly, GO toxicity level varied and highly dependent on time of exposure and dose of the compound [102, 103]. On the other hand, the recent by Saeed et al. showed that Mac-T cells appeared to have tolerance to GO with cell viability were only affected when cells were exposed to GO at concentration higher than required concentration to kills bacteria [103]. Suggesting that this compound has lower toxicity levels and its can be a good potential alternative antimicrobial for treatment of mastitis.

**Table 5:** Antimicrobial activities of graphene-based materials on different pathogenic bacteria associated with mastitis

Bacterial species	Graphene Materials	Concentration	Evaluation Method	Bacterial Inhibition (%)	Reference
*E. coli*	GO	100 μg/mL	Colony Forming Unit (CFU) Count and quantification of ROS, and nucleic acid leakage	89.8%	[102]
*E. coli*	T-rGO	100 μg/mL	CFU Count, and quantification of ROS, and nucleic acid leakage	87.7%	[102]
*S. aureus*	GO	200 µg/mL	CFU Count	90%	[103]
*P. aeruginosa*	GO	62.5 µg/mL	Disk diffusion method of Kirby and Bauer (DDM), and MIC	100%	[104]
*S. aureus*	GO	125 µg/mL	DDM, and MIC	100%	[104]
*S. aureus*	rGO@AgNCs	15.62 µg/mL	DDM, and MIC	100%	[104]
*P. aeruginosa*	rGO@AgNCs	15.62 µg/mL	DDM, and MIC	100%	[104]

### Other alternative approaches

Alternative approaches to mastitis treatment, aside from antibiotics, encompass animal-derived products like lactoferrin [105] and chitosan [106], as well as microbial-derived substances like bacteriocin [26, 107, 108]. Kutila et al. [105] reported that lactoferrin showed similar effectiveness to that of enrofloxacin against *E. coli* isolates. Chitosan based nano formulation exhibited antimicrobial activity against mastitis pathogens in a dose-dependent manner and were able to inhibit biofilm formation [106]. Lactococcal bacteriocin, nisin, lacticin 3147 are some of the bacteriocins effective against various pathogens associated with mastitis [108]. These alternatives have demonstrated significant efficacy in *both* in *vivo* and in vitro experiments.

## Conclusion

Mastitis is a rising threat in the dairy industry associated with economic losses. The ability of mastitis- causing bacteria to develop resistance to commonly used antimicrobials, to form biofilm, invading and surviving with mammary epithelial cells further complicates the problem and renders antibiotics used to cure mastitis ineffective. Addressing this growing challenge requires devising new alternative treatment options. Herbal compounds, bacteriophage therapy, antimicrobial peptides (AMPs), and graphene nanoparticle-based therapy are promising in the treatment of mastitis. This suggests the possibility of using them either alone or in combination with existing antimicrobials for mastitis treatment. Further studies are needed to advance the highlighted alternative options and make them available to farmers .

## Data Availability

All obtained data from this study was included in this manuscript and are available on request from the corresponding author [Shamsaldeen I Saeed].

## Abbreviations

AMPs: Antimicrobial peptides
AMR: Antimicrobial resistance
CBNMs: Carbon-based nanomaterial
CFU: Colony forming unit
DDM: Disk diffusion method of Kirby and Bauer
DPI: Day post-inoculation
GO: Graphene oxide
IMI: Intramammary infection
LA-MRSA: Livestock-associated Methicillin-resistant *Staphylococcus aureus*
MAC-T: Mammary alveolar cells
MBCs: Minimum bactericidal concentrations
MICs: Minimum inhibitory concentrations
MRSA: Methicillin-resistant *Staphylococcus aureus*
MSSA: Methicillin-sensitive *Staphylococcus aureus*
rGO: Reduced graphene
SCC: Somatic cell count
VISA: Vancomycin intermediate *Staphylococcus aureus*
