# Correction: Confronting the complexities of antimicrobial management for *Staphylococcus aureus* causing bovine mastitis: an innovative paradigm

**DOI:** 10.1186/s13620-024-00266-z

**Published:** 2024-03-09

**Authors:** Shamsaldeen Ibrahim Saeed, Nor Fadhilah Kamaruzzaman, Noel Gahamanyi, Thi Thu Hoai Nguyen, Delower Hossain, Ivan Kahwa

**Affiliations:** 1 Nanotechnology in Veterinary Medicine Research Group, Faculty of Veterinary Medicine, Universiti Malaysia Kelantan (UMK), Pengkalan Chepa, 16100 Kelantan, Malaysia; 2 Microbiology Department, Faculty of Veterinary Science, University of Nyala, PO Box 155, Nyala, Sudan; 3 Biology Department, School of Science, College of Science and Technology, University of Rwanda, P.O. Box 3900, Kigali, Rwanda; 4 Microbiology Unit, National Reference Laboratory, Rwanda Biomedical, P.O. Box 7162, Kigali, Rwanda; 5 Research Center for Infectious Diseases, International University, Vietnam National University, Ho Chi Minh City, Vietnam; 6 Department of Veterinary Medicine and Animal Sciences (DIVAS), Università Degli Studi Di Milano, 26900 Lodi, Italy; 7 Department of Medicine and Public Health, Faculty of Animal Science and Veterinary Medicine, Bangla Agricultural University (SAU), Sher-E, Dhaka, 1207 Bangladesh; 8 Udder Health Bangladesh (UHB), Chattogram, 4225 Bangladesh; 9 Department of Pharmacy, Faculty of Medicine, Mbarara University of Science and Technology, P.O Box 1410, Mbarara, Uganda

**Correction** **:** **Ir Vet J 77, 4 (2024)**

**https://doi.org/10.1186/s13620-024-00264-1**

Following publication of the original article [1], the author reported that there is an error in the article title. The word “***Staphyloccous aureus***” should be “***Staphylococcus aureus***”

The incorrect title is: Confronting the complexities of antimicrobial management for *Staphyloccous aureus* causing bovine mastitis: an innovative paradigm

The correct title is: Confronting the complexities of antimicrobial management for *Staphylococcus aureus* causing bovine mastitis: an innovative paradigm

This has been corrected above and the original article has been updated.
